# Olfactory discrimination and identification as prognostic markers of fitness-to-drive in older drivers

**DOI:** 10.1038/s41598-022-26262-3

**Published:** 2022-12-16

**Authors:** Katerina Touliou, Nicos Maglaveras, Evangelos Bekiaris

**Affiliations:** 1 CERTH/ HIT, 57001 Thessaloniki, Greece; 2 School of Medicine, AUTh, 54124 Thessaloniki, Greece

**Keywords:** Neuroscience, Biomarkers, Engineering

## Abstract

The necessity for reliable, standardized and validated fitness to drive assessment tools for older drivers have been highlighted and discussed for over three decades. Existing neuropsychological tests of driving performance are focusing mostly on visuo-spatial attention and executive functioning rather than other senses. Over the last decade, olfactory deterioration has been found to be associated with cognitive decline and predicting transition from mild cognitive impairment to dementia. The AGILE fitness to drive battery is standardized for older drivers. In this study it was adapted to include the olfactory Sniff’ and Stick’s test. The aim was to investigate the value of relevant deficits as predictive markers of driving ability in three driving groups (older drivers with: (a) no impairment (controls), (b) with Mild Cognitive Impairment (MCI) and (c) MCI and other chronic conditions, i.e., comorbidities). So far, no other study has investigated the predictive value of olfactory deficits in driving ability. The findings revealed that discrimination is important for the first year of the examination and as the decline progresses, identification becomes the better olfactory marker. The latter is also evident in the literature. Hence, the results showed that less indicators are required compared to the initial battery. The olfactory markers were dominant over the neuropsychological tests, apart from alertness, for predicting the older driver’s fitness to drive regardless of the presence of cognitive impairment and other chronic conditions.

## Introduction

The necessity for reliable, standardized, and validated fitness to drive assessment tools for older drivers has been highlighted and discussed for over three decades^1–4^. Distinguishing between able and non-able or safe and unsafe older drivers has been a medical screening process for many years. These screening processes are often carried out by physicians using certain tools with no standardized or clear validity on predicting or assessing the older driver’s driving fitness and performance. On top of that there is no consensus on re-assessment standards across countries as well as no consideration for their cognitive and neuropsychological state.

Cognitive decline is associated with higher risk of involvement into accidents and unfit driving performance^5–7^. However, it is difficult to advice older drivers about their ability to drive because it is affected by many factors^8^. Many assessment tools have been proposed, but there is not consensus on which ones to use in everyday medicine practice^9,10^. Most assessment methods rely on on-road tests (i.e., pass-fail system) without alternatives and variations in their accuracy to predict fitness to drive^11,12^. This often happens because of heterogeneity in sample sizes (e.g., small sizes can often increase the random error, cohorts, participants’ impairment types, etc.)^13^.

Existing neuropsychological tests of driving performance are focusing mostly on visuo-spatial attention and executive functioning rather than other senses^14,15^. Over the last decade, olfactory deterioration has been found to be associated with cognitive decline and that it can predict transition from mild cognitive impairment to dementia^16^. However, there are no studies so far considering olfactory deficits as a predictive marker of fitness to drive to older drivers presenting early signs of cognitive impairment or not.

The AGILE EU project (QLRT-2001-00118) proposed a standardized assessment on a European level, using a common battery to evaluate older drivers’ ability to drive based on the older drivers’ needs and requirements and relevant testing scenarios^17^ as well as the inclusion of relevant criteria and assessment methods^18^. As such, the AGILE-based modular stepwise assessment procedure was proposed^19^. The AGILE project outcomes helped to acquire knowledge on older drivers’ physical, cognitive, behavioural and driving interaction issues. This study utilized this standardized fitness to drive protocol and adapted it to further include olfactory tests to investigate their predictive fitness to drive value in older drivers.

The objectives were primarily the following:

- Assess the change in physical, cognitive, neuropsychological aspects and driving skills over a 12-month period (from Phase I to Phase II) in these 3 targeted study groups.
- Develop predictive models of fitness to drive based on the adapted AGILE protocol.

## Results

Both between and within comparisons were performed (i.e., MANOVAs) to investigate significant changes between study groups in Phase I and II and over-time for each group separately. The types, sources and categorisation of each data collected per variable are presented in Table 1. Further binary logistic regression models were fitted to the data to investigate the hypothesis that an older driver is fit or not drive in a preliminary assessment (Tier 1), or an in-depth assessment (Tier 2) based on the scores in the variables considered in each Tier (Table 2) and the addition of 3 olfactory indicators (threshold, identification and discrimination).

**Table 1:** Variables per Tier.

Instrument^1,2^	Category^3^
**Tier 1**	
Trail Making Test A (TMT-A)^1^	time (sec)
Ability Index^1^	score
Tiredness Index^1^	Same as above
Total number of errors in executive control^2^	number of errors
**Tier 2**^2^	
Non-spatial attention	mean reaction time (msec)
Active visual field (central/Total)	Mean reaction time (msec) and number of omissions
Alertness	Mean/ SD of reaction time (msec)
Go/NoGo	Mean reaction time (msec) and number of errors
Distractability/ distractor	Number of omissions
Flexibility	Mean reaction time (msec) and number of errors
**Tier 3**	
Basic driving skills (Part 1) Non-spatial attention (Part 2) Visuo-spatial attention (Part 3) Executive (Part 4)	Mean rating score by two observers (i.e., driving test)
Final decision	Binary/ Fit to drive (1)/ No fit to drive (0)

^1^Pen and paper standardized test or questionnaire.

^2^Computerised neuropsychological assessment (TAP-M).

^3^All variables are continuous unless stated otherwise.

**Table 2:** Pass/ fail percentages based on Tier outcome.

User group	Phase I		Phase II		*P*
	Pass	Fail	Pass	Fail	
Control	67.35% (33)	32.65% (16)	29.41% (10)	70.59% (34)	< .001
MCI	62.75% (32)	37.25% (19)	26.1% (12)	73.9% (34)	< .001
MCI and comorbid conditions	44% (22)	56% (28)	14% (7)	86% (43)	= .001
Total	58% (87)	42% (63)	26.1% (29)	73.9% (111)	< .001

### Pre-screening assessment (Tier 1)

During the first phase of the experiment, the AGILE protocol was implemented in order to evaluate the change in fitness-to-drive across 3 Tiers. The same individuals were tested after a year, to evaluate the change in driving skills and investigate which factors were affected most. All users completed all Tier levels, regardless of failing at a Tier level or not. Eight participants were replaced with matched individuals because they dropped off (N = 6) or they passed away (N = 2). The replacing participants were matched for sex, age, educational level, and group membership.

Tier 1 is estimated based on the Trail Making Test A (TMT-A) score, the ability and tiredness indices of the IADL, as well as the executive control errors recorded by the neuropsychological assessment battery TAP-M.

#### Trail Making Test (TMT-A)

The overall repeated General Linear Model (GLM) was statistically significant (*F*(2, 143) = 3.214, *p *= .043, η^2 ^= .043, observed power = .606) with an observed decrease of 7.02 sec in the completion time, from Phase I (57.54 ± 1.92) to Phase II (50.15 ± 1.68). However, further Bonferroni adjusted comparisons between the group (with adjusted α = .0072), did not reveal any statistically significant differences. The TMT—A scores increased in Phase II, very similarly between the control (x̅ [mean score] = 5.28 ± 2.45) and the MCI group (x̅ = 6.26 ± 2.70), but differently for the MCI & Other Co group (x̅ = 8.30 ± 2.36). All user groups performed ‘better’ in Phase II, but the change was steeper for the ‘MCI and Other Co’ group. Over 65% of participants reported they remembered the test administered from the first time (i.e., Phase I).

#### Ability and Tiredness Index (IADL) and Executive errors (TAP-M)

The ability and tiredness index are calculated from selected question items of Instrument of Activities of Daily Living (IADL). They both reflect the capacity and how tiring every day activities can be for the individual. As MCI participants are still active and able, we did not expect these indices to considerably change compared to the control group. No statistically differences were found among the groups in Phase I and Phase II. No mean index exceeded 1, which means that their perceived functional level is perceived as normal and does not differ among the groups. The same holds true for the executive errors. It appears that a great number of users failed in the Tier 1 tests. The index is calculated based on a logit regression model equation with defined coefficients resulting by AGILE project results^18,20^. The current coefficients are based on the findings of this project, and they were calculated based on multiple studies conducted with older drivers (with no health issues) across many EU pilot sites.

The following equation was applied^18^:

$${\mathbf{1}}/{\mathbf{1}} + {\mathbf{EXP}}( - \left( { - {\mathbf{7}}.{\mathbf{483}} + {\mathbf{0}}.{\mathbf{095}}*{\mathbf{TMT}}\_{\mathbf{A}} \, {\mathbf{7}}.{\mathbf{942}}*{\mathbf{Ablity}}\_{\mathbf{Index}} + {\mathbf{7}}.{\mathbf{959}}*{\mathbf{Tiredness}}\_{\mathbf{Index}} + {\mathbf{0}}.{\mathbf{045}}*{\mathbf{ec}}\_{\mathbf{err4}}} \right)$$

Strict cut-off points aim to narrow down the possibility of false/positives and false/negatives. If the Tier 1 index is below .28, then the user is found to be ‘fit-to-drive’ and no further testing is required. However, almost a third of healthy drivers were found to be unfit to drive (32.7%), even more MCI drivers (37.3%) and an astounding majority of drivers with various co-morbid conditions (56%). This is a ‘frightening’ finding, if we consider that 42% of the whole sample-regardless user group membership- were found unfit to drive from the early pre-assessment part of the driving assessment battery (i.e., Tier 1).

A significant statistical difference was found in Pass/Fail ratio among the three user groups (*χ*^2^(2) = 6.252, *p *= .044). The significance lies in the comparisons between the control group and the MCI group with the comorbid conditions group.

Great deterioration was found to drivers after 1 year from the initial assessment. Based on Tier 1 results, above 70% of drivers failed in this assessment. Specifically, the NP1 score (i.e., the probability index) significantly increased after 1 year, when the same drivers were assessed again (*F*(1 = 42.497, *p < *.001). In particular, the index almost doubled from 1 year to the next (.35 ± .03 and .61 ± .03, respectively). In both cases, the averages are far above the normal cut-off threshold (i.e., .28). The mean difference between the mean indexes is .26. This finding implies a considerable deterioration of driving skills in all groups, including the control group. This might mean that there might an issue with the reliability of Tier 1 because of higher number of false positives. This is further supported by the raters’ assessment in Tier 3 (Table 2). It is important to note, that users were allocated to the control group based on mental and physical functioning and not because of driving profile. Therefore, according to the pre-screening assessment, the following contingency table presents the number of participants who passed and failed Tier 1 in both phases.

#### In-depth assessment (Tier 2)

Even if a participant fails in one of the neuropsychological cluster of tests, namely non-spatial attention (6 tests), visuo-spatial attention (5 tests), executive attention (10 tests), then they automatically failed this Tier. Failure means they failed at least half of the tests in each cluster (i.e., 3 tests in first and second cluster and 5 in the third cluster).

Overall, difference between phases was not statistically significant. 13.8% of drivers failed Tier 2 assessments in Phase I and 11% in Phase II. Overall, almost 3% more participants failed in Phase I compared their assessment 1 year afterwards. This result contradicts Tier 1 results, where significantly more participants failed in the second phase when compared to the first one. The comparison of overall percentages is not statistically significant (*p *> .05).

Only 4.1% (N = 2) of the control group failed Tier 2 when compared to 8.2% (N = 4) in Phase I. However, the difference was not statistically significant (*p* > .05). 25% participants from the MCI group failed in Phase II and only 9.4% in Phase I. The difference is statistically significant (χ^2^(1)* = *4.477, *p* = .034). 11.8% of the comorbidity group failed in Phase I and 14.8% in Phase II. The difference is not statistically significant (*p *> .05). The next step is to identify in which neuropsychological aspects each group failed from the 3 dimensions included in Tier 2 and identify if any commonalities exist among groups.

##### Phase I

Statistically significant more omissions in the peripheral visual field were made by the MCI & Other Co group when compared to the control (no cognitive impairment) group (mean difference = 12 events, Bonferroni adjustment, *p < *.001) and the mean visual scanning time for critical events was much shorter for the control group when compared to the MCI group (mean difference = 932 msec, Bonferroni adjustment, *p < *.001).

##### Phase II

Mean alertness (mean difference: 42* ± *15.23 msec) and standard deviation of alertness (23.23 ± 8.21 msec) is statistically significantly lower in the control group when compared to the MCI & Other Co group. Overall, the control group was more attentive and alert when compared to the drivers with MCI and MCI and other comorbidities groups.

### Olfactory deterioration

Three levels of change in olfactory ability were measured: a) threshold, b) discrimination, and c) identification. Multivariate Analysis of Variance (MANOVA) tests were performed to investigate differences among the three groups and corrected with Bonferroni post-hoc tests.

#### Threshold

A two-way repeated measures ANOVA was performed to evaluate the difference in olfactory threshold levels among the three groups and across time (i.e., 1 year passed from Phase I to Phase II). The assumptions related to the application of normal distribution statistical tests were not violated. Overall, statistically significant decrease of 0.478 in mean threshold scores was found a year after the first test, from 5.468 (SE = 0.184) to 4.999 (SE = 0.194) threshold (*F*(*1, 144*) = 4.868, *p* = 0.029, η^2^ = 0.033, Observed power = 0.592). Likewise, significant difference was found in Phase I (*F*(*2, 145*) = 4.881, *p* = 0.009, η^2^ = 0.63, Observed power = 0.797) and Phase II (*F*(*2, 144*) = 16.908, *p* < 0.001, η^2^ = 0.192, Observed power = 1), respectively.

##### Phase I

**Figure 1:**
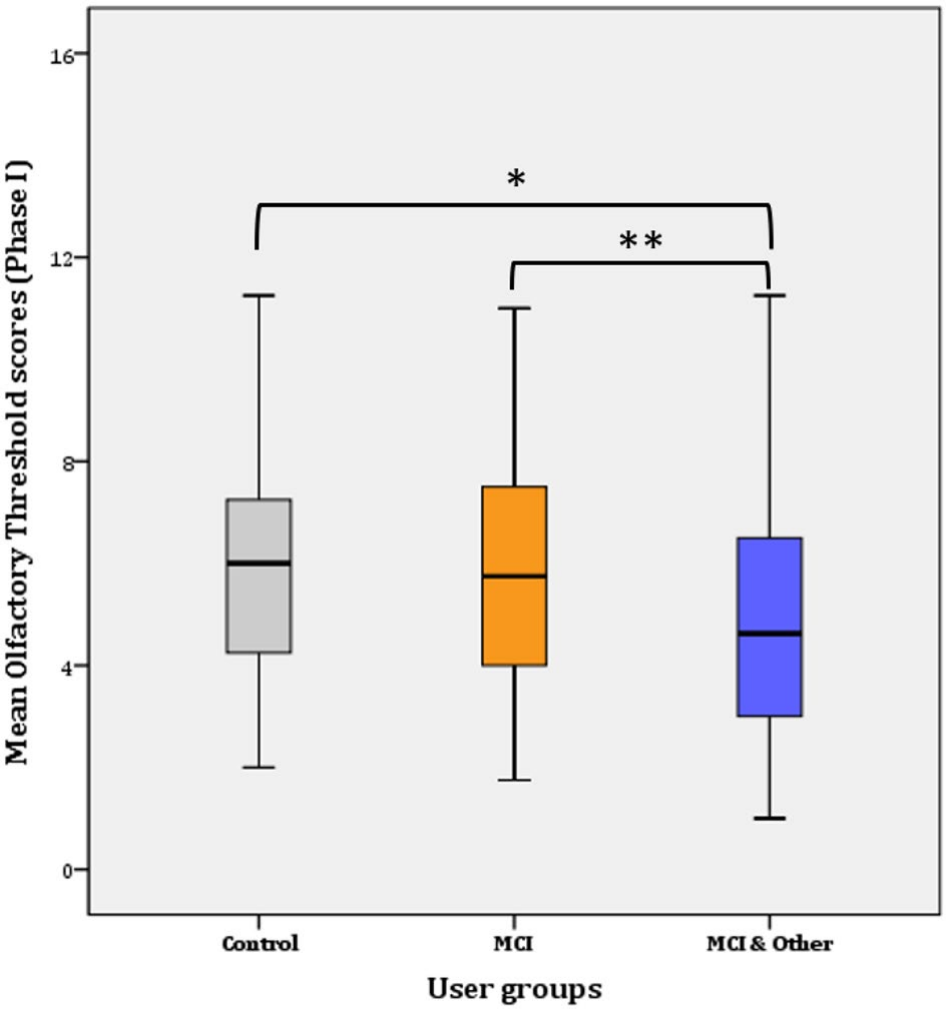
Mean Threshold scores per user group (Phase I).

Further Bonferroni adjustment revealed that the significance lies in the mean difference (1.283 ± 0.447) between the control and the MCI & Other Co group (*p* = 0.014*) and the MCI and the MCI & Other Co group (1.956 ± 0.376; *p* < 0.001**). The difference between the control and the MCI (0.22 ± 0.458) groups was not statistically significant (*p* > 0.017; adjusted for pairwise comparisons) (Fig. 1).

##### Phase II

**Figure 2:**
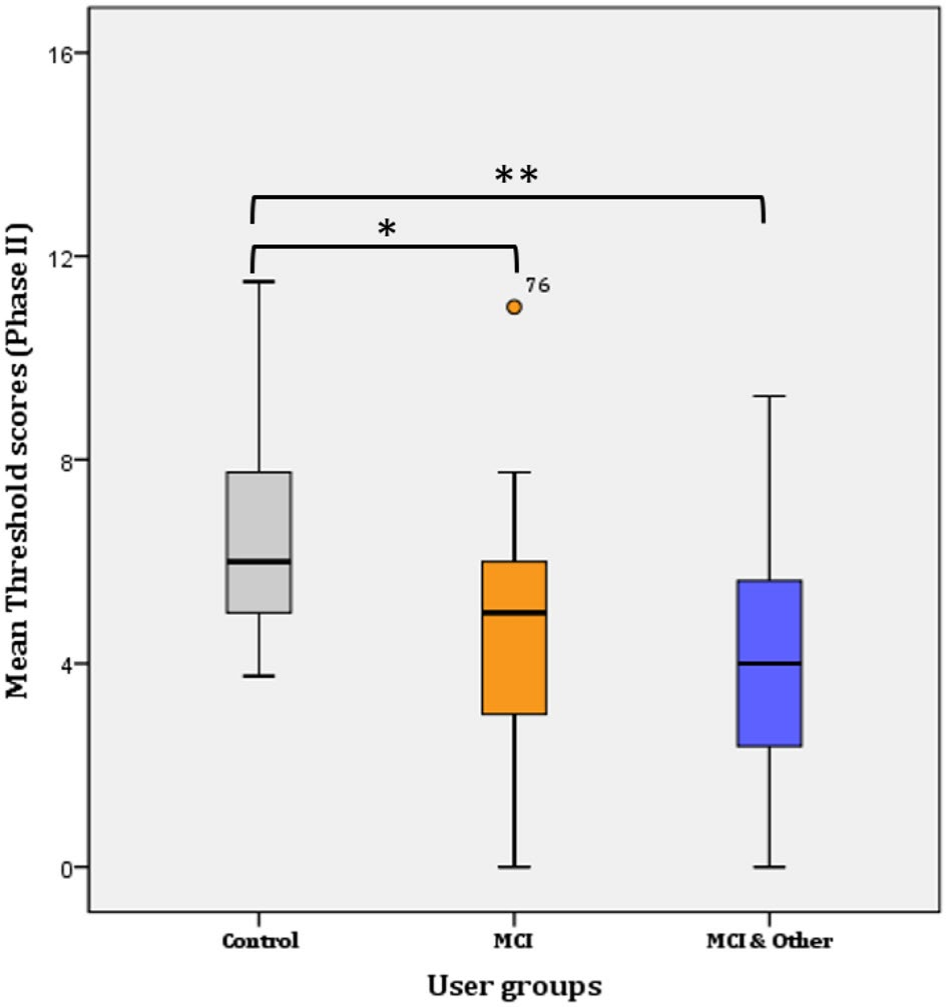
Mean Threshold scores per user group (Phase II).

Further Bonferroni adjustment revealed that the significance lies in the mean difference (2.022 ± 0.482) between the control and the MCI group (*p* < 0.001*) and the control and the MCI & Other Co group (2.686 ± 0.477; *p* < 0.001**). The difference between the MCI and the MCI & Other Co (0.664 ± 0.467) groups disappeared and it was not statistically significant (*p* > 0.017; adjusted for pairwise comparisons) (Fig. 2).

Overall, mean threshold scores increased (by 0.618) in the control group from Phase I (5.95 ± 0.315; CI 5.315–6.585) to Phase II (6.568 ± 0.303; CI 5.957–7.179). Standard Error is sometimes estimated instead of Standard Deviation, as it is a better depiction of the precision of the mean measurement and mean differences are of interest for the comparisons at hand. The difference is not statistically significant (*F*(1, 44) = 3.396, *p* = 0.072), but a trend for increase is evident. On the contrary, the mean threshold scores significantly decreased in the MCI (mean decrease = 1.184; *F*(1, 48) = 17.427*, p* < 0.001), but not in the MCI & Other Co group (mean decrease = 0.843; *F*(1, 50) = 17.427*,* *p* < 0.087). The latter tends towards significance but because an increase was found in the ‘younger’ age group (i.e., 51–60 years), statistically significant decrease was not obtained.

As Konstantinidis and colleagues^21^ found similar norms with the German population, it is safe to infer that probably applying the current thresholds resulting from the same population will be similar and as such the same norms were applied for the study sample. Most participants were below the norm regardless group membership in both Phases, with the control group having more participants scoring below the norm (N = 33) than the MCI group (N = 30), but less than the MCI & Other Co group (N = 42). The performed χ^2^ was marginally non-significant (*p* = 0.06). Likewise, most participants are below the norm in Phase II with the difference becoming significant (*χ*^2^ (2) = 12.632, *p* = 0.002). However, 13% more participants were above the norm in the control group in Phase II. Statistically significant different proportions with Bonferroni adjusted α level at 0.0083 were found in the control and the MCI & Other Co groups (*p* = 0.0009 and *p* = 0.0061, respectively).

#### Discrimination

Overall, discrimination ability significantly declined from Phase I (13.18 ± 0.23) to Phase II (10.86 ± 0.33) (*F*(1, 147) = 56.559, *p* < 0.001, *η*^2^ = 0.278, observed power = 1).

##### Phase I

Taking into consideration the user group membership in the General Linear Model (GLM), the statistical significance pertains (*F*(2, 145) = 7.740, *p* = 001, *η*^2^ = 0.096, observed power = 0.946). Further comparisons (Bonferroni adjusted α level = 0.017) revealed significant difference between the control and the MCI & Other Co group (${\bar{\text{x}}}$ = 2.13, *p* < 0.001*) and no significant difference between the control group and the MCI group (${\bar{\text{x}}}$ = 1.42, *p* > 0.017; NS). Similarly, the difference between the MCI and the MCI and Other Co group was not statistically significant (${\bar{\text{x}}}$ = 0.704, *p* > 0.17; NS).

##### Phase II

**Figure 3:**
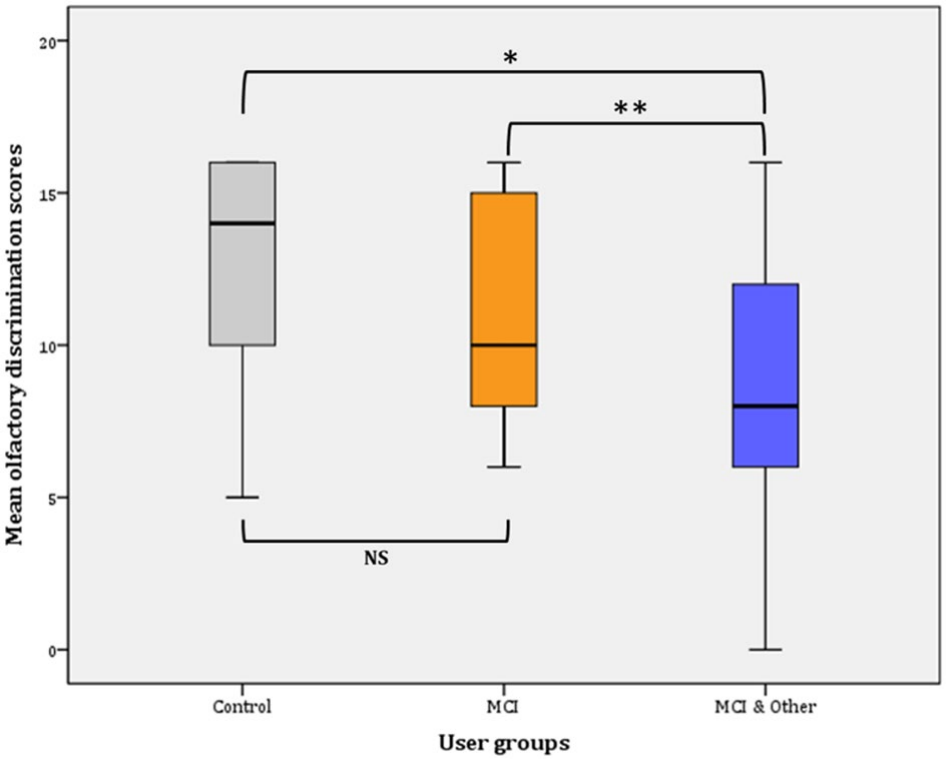
Mean olfactory discrimination scores (Phase II).

Taking into consideration the user group membership in the General Linear Model (GLM), the statistical significance pertains (*F*(2, 145) = 13.947, *p* < 0.001, *η*^2^ = 0.161, observed power = 0.998). Further comparisons (Bonferroni adjusted α level = 0.017) revealed significant difference between the control and the MCI & Other Co group (${\bar{\text{x}}}$ = 3.889, *p* < 0.001*) and no significant difference between the control group and the MCI group (${\bar{\text{x}}}$ = 1.848, *p* > 0.017; NS). Similarly, the difference between the MCI and the MCI and Other Co group was marginally statistically significant (${\bar{\text{x}}}$ = 2.041, *p* = 0.016**) (Fig. 3).

Pairwise comparisons among the user groups over time, showed that significant differences were found between the control group and the experimental groups, i.e., marginal for the MCI (${\bar{\text{x}}}$ = 1635, *p* = 009**) and greater when compared to the MCI and Co group (${\bar{\text{x}}}$ = 3.007, *p* < 0.001*). However, the difference between the two experimental groups was not statistically significant (${\bar{\text{x}}}$ = 1372, *p* = 0.028; NS). The Bonferroni adjusted α was set at 0.0083.

##### Phase I

Statistically significant differences in the proportions of participants in each user group were found (*χ*^2^(2) = 20.438, *p* < 0.001). Statistically significant different proportions -with Bonferroni adjusted α level at 0.0083- were found in the control and the MCI & Other Co groups (*p* = 0.0003 and *p* = 0.000003, respectively).

##### Phase II

Statistically significant differences in the proportions of participants in each user group were found (*χ*^2^(2) = 13.948, *p* = 0.001). Statistically significant different proportions with Bonferroni adjusted α level at 0.0083 were found in the control and the MCI & Other Co groups (*p* = 0.0005 and *p* = 0.003, respectively).

#### Identification

Overall, identification ability significantly declined from Phase I (9.93 ± 0.17) to Phase II (8.99 ± 0.19) (*F*(1, 129) = 56.559, *p* < 0.001, *η*^2^ = 0.111 observed power = 0.986).

##### Phase I

**Figure 4:**
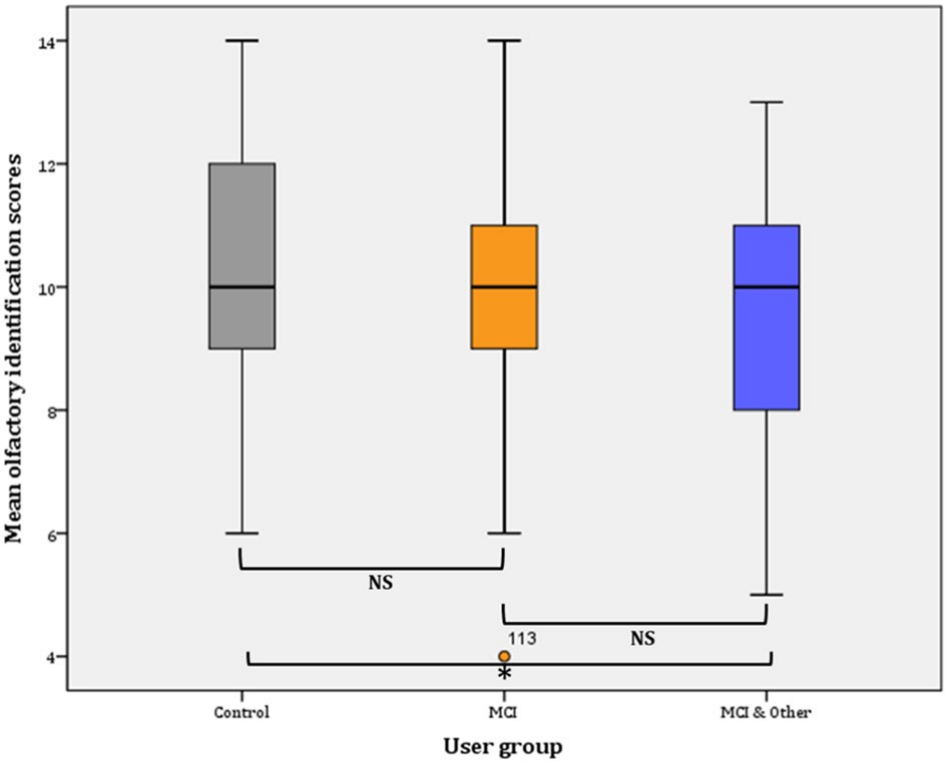
Mean olfactory identification scores (Phase I).

Taking into consideration the user group membership between factors in the General Linear Model (GLM), the statistical significance pertains (*F*(2, 137) = 7.818, *p* = 001, *η*^2^ = 0.102, observed power = 0.948). Further comparisons (Bonferroni adjusted α level = 0.017) revealed significant difference between the control and the MCI & Other Co group (${\bar{\text{x}}}$ = 1.33, *p* < 0.001*) and no significant difference between the control group and the MCI group (${\bar{\text{x}}}$ = 0.748, *p* > 0.05; NS). Similarly, the difference between the MCI and the MCI and Other Co group was not statistically significant (${\bar{\text{x}}}$ = 0.585, *p* > 0.05; NS) (Fig. 4).

**Figure 5:**
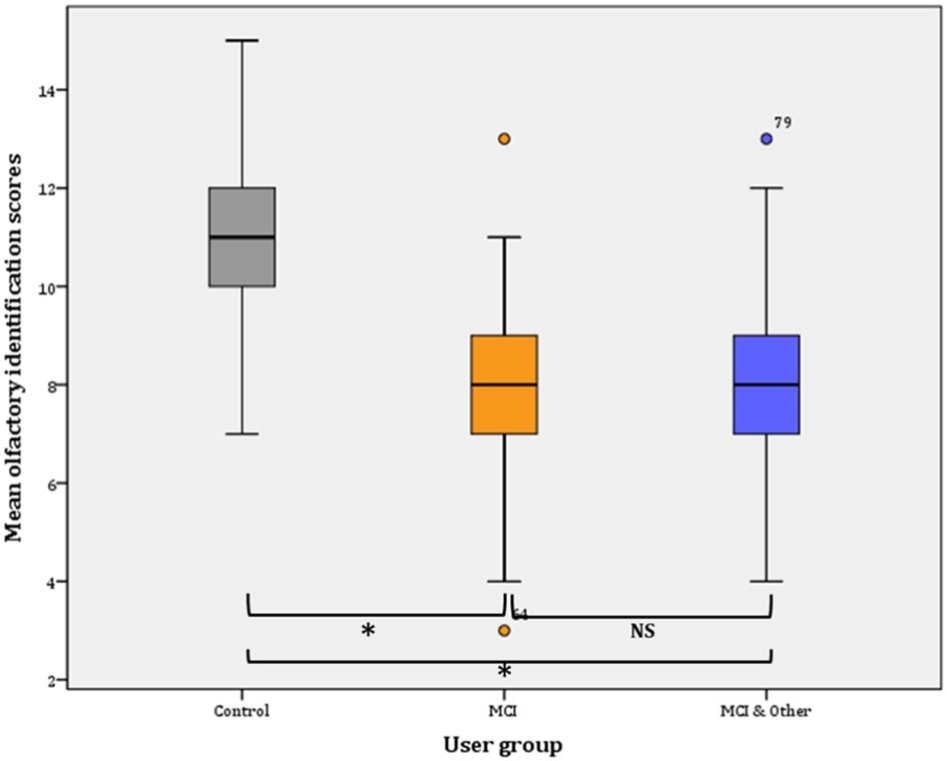
Mean olfactory identification scores (Phase II).

##### Phase II

Taking into consideration the user group membership in the General Linear Model (GLM), the statistical significance again pertains (*F*(2, 145) = 38.750, *p* < 0.001, *η*^2^ = 0.348, observed power = 1). Further comparisons (Bonferroni adjusted α level = 0.017) revealed significant difference between the control and the MCI & Other Co group (${\bar{\text{x}}}$ = 2.937, *p* < 0.001*) and significant difference between the control group and the MCI group (${\bar{\text{x}}}$ = 2.978, *p* < 0.001*). On the contrary, the difference between the MCI and the MCI and Other Co group was not statistically significant (${\bar{\text{x}}}$ = 0.041, *p* = . > 0.05; NS) (Fig. 4).

Overall, mean identification scores slightly decreased (by 0.359) in the control group from Phase I (10.33 ± 0.329; CI 9.667–11) to Phase II (9.974 ± 0.395; CI 9.175–10.774). The difference is not statistically significant (*F*(1, 38) = 0.749, *p* = 0.392). On the contrary, the mean threshold scores significantly decreased in the MCI group (mean decrease = 1.563; *F*(1, 47) = 16.401*, p* < 0.001). Likewise, statistically significant decrease was observed for the MCI & Other Co group (mean decrease = . 0.887; *F*(1, 52) = 5.025*, p* < 0.029) (Fig. 5).

##### Phase I

Statistically significant differences in the proportions of participants in each user group were marginally found (*χ*^2^(2) = 6.125, *p* = 0.047). Statistically significant different proportions -with Bonferroni adjusted α level at 0.0083-were not found for any pairwise comparison.

##### Phase II

Similarly, overall, statistically significant differences in the proportions of participants in each user group were found (*χ*^2^(2) = 6.645, *p* = 0.036). Statistically significant different proportions with Bonferroni adjusted α level at 0.0083 were not found in any of the pairwise comparisons.

### Predictive models of fitness to drive

The types, sources and categorisation of each data collected per variable are presented in Table 2. Further binary logistic regression models were fitted to the data to investigate the hypothesis that an older driver is fit or not to drive following a preliminary assessment (Tier 1) or an in-depth assessment (Tier 2), based on the scores in the variables considered in each Tier (Table 1) and the addition of 3 olfactory indicators (threshold, identification and discrimination) and taste ability. In addition, the logistic regressions analyses were performed to investigate which indicators can predict fitness to drive in the 3 groups. The predictor variables were tested a priori to verify that there was no violation of the linearity of the logit. The predictor variables that were included in the logit regression models are only the ones that they were found to contribute to the model, as a stepwise addition was made. Olfactory variables were added to the existing variables for Tier 1 and Tier 2. A stepwise addition of variables showed that only the ones that are shown below are significant.

The regression model is expressed with an equation where in the logit model the log odds of the outcome is modelled as a linear combination of the predictor variables. A popular statistical technique to predict binomial outcomes (y = 0 or 1) is Logistic Regression. Logistic regression predicts categorical outcomes (binomial / multinomial values of y), whereas linear Regression is good for predicting continuous-valued outcomes. The predictions of Logistic Regression (henceforth, LogR in this article) are in the form of probabilities of an event occurring, ie the probability of y = 1, given certain values of input variables x. Thus, the results of LogR range between 0–1.

In **Tier 1**, the results showed for the first assessment that the logit model is the following fitted for one predictor model with constant and unstandardised Beta weight coefficients:

$${\text{Predicted}}\,{\text{logit}}\,{\text{of}}\,\left( {\text{Fitness - to - drive}}_{{{\text{All}}}} \right)_{{{\text{Tier1}} - {\text{Phase}}\,{\text{I}}}} = {1}.{174} + \left( {.{27}0} \right)*{\text{OLFACTORY}}\,{\text{DISCRIMINATION}}$$

In **Tier 1 and Model of Phase I**, the predictor variable included in the logit regression model was found to be olfactory discrimination and this one only contributed to the model. The unstandardised Beta weight for the Constant; *B* = 1.174, *SE* = 0.196, *Wald* = 35.804, *p* < 0.001. The unstandardised Beta weight for the Predictor; *B* = 0.270, *SE* = 0.115, *Wald* = 5.481, *p* = 0.019. The estimated odds ratio favoured an increase of 13.1%, 95% CI (1.045–1.642) for fitness to drive for every one unit increase of olfactory discrimination ability.

Accordingly, the model for MCI drivers, it is adjusted as follows:

$${\text{Predicted}}\;{\text{logit}}\;{\text{of}} \;\left( {\text{Fitness}}-{\text{to}}-{\text{drive}}_{{{\text{MCI}}}} \right)_{{{\text{Tier1}} - {\text{Phase I}}}} = \, - {2}.{771} + \, \left( {0.{383}} \right)*{\text{OLFACTORY}}\; {\text{DISCRIMINATION}}$$

Further, the model for MCI drivers with comorbidities, it is adjusted as follows:

$${\text{Predicted}}\;{\text{logit}}\;{\text{of}}\;\left( {\text{Fitness}}-{\text{to}}-{\text{drive}}_{{{\text{MCI}} + {\text{Co}}}} \right)_{{{\text{Tier1}} - {\text{Phase}}\;{\text{I}}}} { = 6}.{326} + \left( { - {18}.{46}0} \right)*{\text{TIREDNESS}}\;{\text{INDEX}} + \left( {0.{566}} \right)*{\text{TASTE}} + \left( {0.{514}} \right)*{\text{OLFACTORY}}\;{\text{THRESHOLD}}$$

For the second assessment that followed after 1 year (Phase II) for each participant the model changes into the following:

$${\text{Predicted}}\;{\text{logit}}\;{\text{of}}\;\left( {\text{Fitness}}-{\text{to}}-{\text{drive}}_{{{\text{All}}}} \right)_{{{\text{Tier1}} - {\text{Phase II}}}} = { 1}0.{3}0{8} + \, \left( { - .{2}0{8}} \right)*{\text{AGE }} + \, \left( {.{157}} \right) \, *{\text{OLFACTORY}}\; {\text{IDENTIFICATION}}$$

In **Tier 1 and Model of Phase II**, the predictor variables included in the logit regression model were found to be olfactory identification, but also age to contribute to the model. The unstandardised Beta weight for the Constant; *B* = − 1.414, *SE* = 0.215, *Wald* = 43.403, *p* < 0.001. The unstandardised Beta weight for the Predictors; Age: *B* = − 1.414, *SE* = 0.215, *Wald* = 43.403, *p* < 0.002 and Olfactory identification: *B* = 0.159, *SE* = 0.085, *Wald* = 3.503, *p* = 0.061 The estimated odds ratios favoured an increase of 77.5%, 95% CI (66.21–93.70) for fitness to drive for every one unit increase of olfactory identification ability for age and for smell identification 11.72% 95% CI (0.993–1.384).

However, when controlling for age (i.e., removing part of the sample that creates the age statistical difference), the model takes a different shape and only the importance of olfactory deficit in odour remains, as shown in the logit model below. The unstandardised Beta weight for the Constant; *B* = − 3.211, *SE* = 1.695, *Wald* = 3.588, *p* = 0.05. The unstandardised Beta weight for the Predictor; Olfactory identification: *B* = 0.237, *SE* = 0.086, *Wald* = 7.641, *p* = 0.006. The estimated odds ratios favoured an increase of 24.3%, 95% CI (1.071–1.499) for fitness to drive for every one unit increase of olfactory identification ability for smell identification 12.67%, 95% CI (10.22–14.34).

$${\text{Predicted}}\;{\text{logit}}\;{\text{of}}\;\left( {\text{Fitness}}-{\text{to}}-{\text{drive}}_{{{\text{All}}\;{\text{adjusted}}\;{\text{Age}}}} \right)_{{{\text{Tier1}} - {\text{Phase}}\;{\text{II}}}} = - {3}.{211} + \left( {0.{237}} \right)*{\text{OLFACTORY}}\;{\text{IDENTIFICATION}}$$

The model for the control group changes as the following, with the executive errors in the neuropsychological assessment (part of TAP-M) being the predictor of fitness to drive in older drivers. The unstandardised Beta weight for the Constant; *B* = − 3. 115, *SE* = 4.556, *Wald* = 0.468, *p* > 0.05. The unstandardised Beta weight for the Predictor; Olfactory executive errors: *B* = − 0.060, *SE* = 0.028, *Wald* = 4.604, *p* = 0.032. The estimated odds ratios favoured an increase of 22.53%, for fitness to drive for every one unit increase of olfactory identification ability for smell identification 9.42%, 95% CI (0.892–0.995). It is important to note thought that this overall model was not statistically significant and therefore a function is not provided.

Likewise, the model for MCI and MCI & Other Co groups showed a trend towards significance (e.g., for MCI group *p* = 0.09) for olfactory threshold, but not as the overall model.

For **Tier 2**, the results showed that the logit model is the following for the first assessment:

In **Tier 2 and Model of Phase I**, the predictor variables included in the logit regression model were found to be olfactory discrimination, but also age to contribute to the model. The unstandardised Beta weight for the Constant; *B* = 4.795, *SE* = 2.942, *Wald* = 2.656, *p* = 0.103. The unstandardised Beta weight for the Predictors; Age: *B* = − 0.107, *SE* = 0.039, *Wald* = 7.415, *p* = 0.006 and Olfactory discrimination: *B* = 0.384, *SE* = 0.133, *Wald* = 8.355, *p* = 0.004 The estimated odds ratios favoured an increase of 89.9% for fitness to drive for every one unit increase of fitness ability for age and for smell discrimination 14.68% 95% CI (0.993–1.384).

$${\text{Predicted}}\;{\text{logit}}\;{\text{of}}\;\left( {\text{Fitness}}-{\text{to}}-{\text{drive}} \right)_{{\text{Tier2}} - {\text{Phase}}} \; {\text{I}} = {4}.{795} + \left( {.{384}} \right)*{\text{DISCRIMINATION}} + \left( { - .{1}0{7}} \right)*{\text{AGE}}$$

Likewise, when controlling for age, the above model amends as follows:

$${\text{Predicted}}\;{\text{logit}}\;{\text{of}}\;\left( {\text{Fitness}}-{\text{to}}-{\text{drive}} \right)_{{{\text{Tier2}} - {\text{Phase}}\;{\text{I}}\;{\text{All}}\;{\text{Adjusted}}\;{\text{Age}}}} = {2}.0{18} + \left( {.{293}} \right)*{\text{DISCRIMINATION}}$$

The unstandardised Beta weight for the Constant; *B* = 2.018, *SE* = 2.966, *Wald* = 0.463, *p* = 0.026. The unstandardised Beta weight for the Predictor; Olfactory discrimination *B* = 0.293, *SE* = 0.132, *Wald* = 4.940, *p* = 0.026. The estimated odds ratios favoured an increase of 75.21% for fitness to drive for every one unit increase of fitness ability for age and for smell discrimination 14.40% 95% CI (1.035–1.735).

For the subsequent assessment after 12 months the model is the following:

$${\text{Predicted}}\;{\text{logit}}\;{\text{of}}\left( {\text{Fitness}}-{\text{to}}-{\text{drive}}\right)_{{{\text{Tier2}} - {\text{Phase}}\;{\text{II}}}} = {11}.{988} + \left( {.{173}} \right)*{\text{IDENTIFICATION}} + \left( { - .{276}} \right)*{\text{AGE}} + \left( {.00{9}} \right)*{\text{ALERTNESS}}$$

In **Tier 2 and Model of Phase II**, the predictor variables included in the logit regression model were found to be olfactory identification, but also age and alertness to contribute to the model. The unstandardised Beta weight for the Constant; *B* = 11.988, *SE* = 4.956, *Wald* = 5.852, *p* = 0.016. The unstandardised Beta weight for the Predictors; Age: *B* = -0.276, *SE* = 0.078, *Wald* = 12.622, *p* < 0.001, Olfactory identification: *B* = 0.173, *SE* = 0.077, *Wald* = 5.004, *p* = 0.025 The estimated odds ratios favoured an increase of 75.9%, 95% CI (1.011–1.460) for fitness to drive for every one unit increase of fitness ability for age and for smell identification 11.88% 95% CI (0.962–1.323) and alertness *B* = 0.009, *SE* = 0.004, *Wald* = 5.639, *p* = 0.018, with increase of 10.09%.

Controlling for age, this model changes into the following:

$${\text{Predicted}}\;{\text{logit}}\;{\text{of}}\,\left( {\text{Fitness - to - drive}} \right)_{{{\text{Tier2}} - {\text{Phase}}\;{\text{II}}\;{\text{Control}}\;{\text{for}}\;{\text{Age}}}} {\text{Age}} = - {1}.{382} + \left( {.{222}} \right)*{\text{IDENTIFICATION}} + \left( {.0{23}} \right)*{\text{ALERTNESS}}$$

The unstandardised Beta weight for the Constant; *B* = -1.382, *SE* = 2.726 *Wald* = 0.257, *p* = 0.612. The unstandardised Beta weight for the Predictors; Olfactory identification: *B* = 0.222, *SE* = 0.085, *Wald* = 6.782, *p* = 0.009 The estimated odds ratios favoured an increase of 25.1% for fitness to drive for every one unit increase of fitness ability for smell identification 12.49% 95% CI (1.057–1.476) and alertness *B* = 0.009, *SE* = 0.006, *Wald* = 2.533, *p* = 0.012, with increase of 10.91%.

## Discussion

Roalf and colleagues^22^ conducted a meta-analysis of 31 articles and found that MCI patients demonstrate deficits in smell that are robust, and identification is primarily impaired in MCI and that is also the most prevalent sensory deterioration in AD^22^. Similarly, a recent meta-analysis of studies investigating olfactory deterioration in MCI and AD patients, found that identification is often more profoundly impaired in AD rather than MCI, meaning that it can identify the risk of people progressing from MCI to AD and thus can be utilised as a prognostic marker^23^.

It appears that discrimination is important for the first year of the examination and as the decline progresses, identification becomes the better olfactory marker, as it is evident also in the literature^24,25^. In comparison to the AGILE protocol and the regression model, the results in this project showed that less indicators are required with olfactory markers being dominant over the Instruments of Activities of Daily Living (IADL) questionnaire, the TMT-A test, and the neuropsychological battery for predicting the older driver’s fitness to drive regardless of the presence of cognitive impairment and other chronic conditions. The pattern remains unchanged from Tier 1 to Tier 2 assessment with olfactory dimensions appearing to be a stronger predictor of fitness to drive than the other variables, apart from alertness in neuropsychological assessment. The identification score is correlated with neuropsychological processes like visuospatial attention and episodic memory, but its effect prevails over neuropsychological variables in this study.

Furthermore, deterioration in olfactory identification has been associated not only with the transition from MCI to AD, but also with faster cognitive deterioration^26,27^. Olfactory identification may even precede cognitive impairment because of findings in early pathological analyses of the cohorts found strong relation between the appearance of neurofibrillary tangles in the entorhinal cortex and CA1 hippocampus regions of the central olfactory system, identified often as the first site of clinicopathological alterations because of AD^27–29^).

Although participants with acute respiratory infections the last month, nasal surgery, smoking, chronic sinusitis COVID, and obstructive pulmonary disease as well as other chronic diseases that may affect the sense of smell were excluded from the study there are other known and potentially unknown factors that might affect the result of the administered olfactory test. Replication of the study with another sample will allow for control for revealing too many predictive factors in a small sample is often closely related to a caveat known as capitalisation of chance^30^. According to MacCallum and colleagues^30^, large parts of the associations between predictive variables and the outcome about fitness of the driver is because of random error in the overall error estimation.

There is still no consensus on how to evaluate fitness to drive in everyday clinical practice, although many assessment batteries and tools have been developed, mainly because the predictive validity is considerably variant, and accuracy very often cannot replicate the decisions of the road instructors/ assessors^11^. The issue might not be with the assessment tools, but with the diversity in symptoms, sample characteristics, sizes and types of decline and impairment^13^.

However, in comparison to other existing protocols that are mostly based on medical and neuropsychological assessment, this assessment is short and can be part of a larger protocol that does not necessarily focuses on fitness to drive, but also to other conditions (e.g., SARS-Co-2 detection, other neurodegenerative diseases like Parkinson’s, etc.).

Until now olfactory deficits have been a well-known biomarker of neurodegeneration and is regarded as a sign of pathological onset of AD. However, these findings support that olfactory deficit can be a marker of fitness to drive in older drivers with MCI and other chronic conditions. Additionally, the results show that olfactory markers may be stronger predictors than traditional neuropsychological tests, which are time and effort consuming compared to a simple and fast smell test.

Olfactory assessment offers multi-assessment purposes (effective, fast, and cost-effective). In a COVID era, the utilization of olfaction as an instrument of driving ability may allow for multiuse of one battery for more than one application saving not only time but also resources.

One current limitation though that limits the direct application of the findings is the fact that the predictions are based on estimated (derivation set) than on a new data set (validation set). External validation of a prediction model on an independent data set is an important step before clinical application is even considered^30^.

## Methods

### Participants

The sample was drawn from community dwelling adults in the city of Thessaloniki and surrounding regional Thessaloniki Prefecture, Greece, who were recruited primary and tertiary care clinics, geriatricians. Recruitment targeted individuals with mild memory concerns, as well the general community of older drivers, with the aim of obtaining a Mild Cognitive Impairment (MCI) enriched sample as well as older drivers with MCI. We excluded participants who reported a prior diagnosis of dementia (n = 8).

Of 153 respondents, 145 participated and met the criteria for this study. The age-range was 56–84 (mean = 67.35, SD = 6.29, 29% female). MCI diagnosis was performed by an affiliated neurologist according to Petersen’s criteria, i.e., based on: a) memory complaints, b) normal activities of daily living, c) normal general cognitive functioning, d) objective impairment in one area of cognitive functioning as evidence by scores of >1.5 SD of age-appropriate norms (b) or abnormal memory function of age (c), e) no dementia^18,20^. The sample comprised 145 older active drivers divided in three groups: a) Mild Cognitive Impairment (range = 56–83, mean = 66.83, SD = 6.17)—MCI Group, b) Mild Cognitive impairment and other co-morbid conditions (range = 58–84, mean = 69.52, SD = 6.16)—MCI+Co Group and c)no Mild Cognitive Impairment and no other chronic conditions that could affect their olfaction, gustation and driving performance (range = 58–83, mean = 65.44, SD = 5.35)—Control Group. Participants had no evidence of cognitive impairment or psychiatric disorder at the time of clinical and neuropsychological assessment.

Patients who had previously been diagnosed as having smell-and taste-related disease and/or who had taken anti-dementia drugs for AD and MCI and/or who cannot be performed olfactory tests due to severe cognitive dysfunction were excluded. The following clinical and demographic characteristics (Table 3) were assessed: age, sex, smoking, drinking habits, medications, and the presence of hypertension, hyperlipidaemia, diabetes mellitus and other chronic conditions. The MCI+Co group comprised 27 participants with cardiovascular conditions, 14 Metabolic/ endocrinological conditions, and 10 with other conditions (e.g., respiratory, gastrointestinal, myoskeletical), where the numbers were too small to form a group. Within-group comparisons demonstrated that no significant differences were found for any of the dependent variables and therefore the results represent all the members of this group Control-MCI+Co; F(2)=5.69, *p*=.004, Bonferroni adjusted. However, this result should be treated with caution as different distribution of chronic conditions or another group could yield different outcomes. This is only important for this sample and cannot be generalized.

**Table 3:** Characteristics of the participants*.*

Characteristics	MCI (*n* = 48)	MCI + Co (*n* = 52)	Control (*n* = 45)	*p*-value
**Age**	66.83 (6.17)	69.52 (6.16)	65.44 (5.35)	*
**Education**				
Primary	18.9%	21.2%	10.6%	NS
Secondary	47.2%	48.1%	55.3%	NS
Higher	17%	15.4%	10.6%	NS
Other	17%	15.4%	23.4%	NS
**Employment status**				
Retired	86.8%	88.5%	81.6%	NS
Still working	11.3%	11.5%	17.8%	NS
**Marital status**				
Partner	54.7%	57.7%	59.6%	
Family	28.3%	36.5%	23.4%	
Alone	17%	5.8%	14.9%	
Friends	–	–	2.1%	
Driving experience (years)	36.98 (9.00)	39.02 (8.37)	36.53 (1)	NS
Driving frequency (km driven per month)	459.09	714.6	621.91	
**MMSE**				
1st session	23.8 (3.5)	23.9 (3.5)	28.33 (2.8)	
Follow-up session	22.3 (3.2)	22.58 (3.3)	29.22 (0.8)	

MMSE, Mini-mental state examination; MCI, mild cognitive impairment; NS, Non-Statistically significant.

### Design

**Figure 6:**
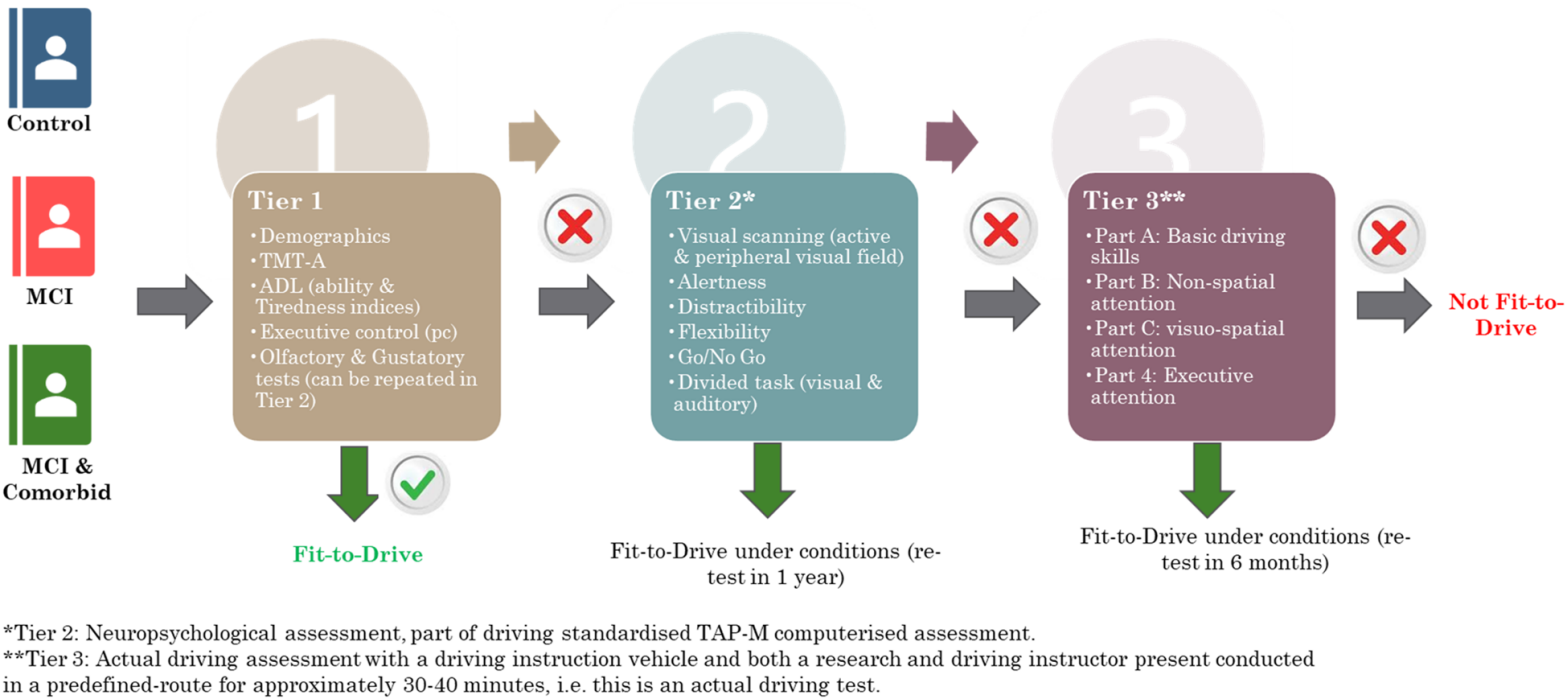
AGILE-adapted assessment procedure.

This is a 2 × 3 repeated measures (within-participants) design. Independent variables are (the presence or not of cognitive impairment) and the fitness to drive. Dependent variables are the following artefacts that are resulting from clusters of indicators and are: a) neuropsychological functioning, b) fitness-to-drive (i.e., driving performance), and c) olfactory functioning. The procedure was conducted by all participants in Phase I and then after 1 year (Phase II) to investigate any changes in the detailed procedure is discussed in the next sub-sections. Figure 6 is an overview of the proposed AGILE assessment procedure for elderly drivers adapted for this study with the addition of olfactory tests.

### Procedure

The research protocol was approved by the Human Research Ethics Committee of Aristotle University and of the Ethics Committee of the Centre for Research and Technology Hellas (CERTH). We confirm that all research was performed in accordance with then national and organizational guidelines and regulations. Informed consent was obtained from all participants. The study was performed in accordance with the Declaration of Helsinki. The research protocol was explained to the patients and/or relatives, and informed consent for their participation was obtained. The study was completed before GDPR adoption, however, any data processing after 25th of May 2018 was performed according to the new directive requirements. No personal data were stored or processed. The recruiter was blind to the experiments and was not part of the experimental process. The researchers who conducted the study had no information about the participant about their personal details and their group membership.

The AGILE protocol was applied that is described in the following figure. This protocol is stepwise and modular. The overall AGILE assessment procedure involves three tiers. A pre-screening and screening are performed in a quiet room in the first tier (Tier 1). In the second Tier (Tier 2) persons failing the screening battery undergo an in-depth diagnostic neuropsychological assessment at a specialised centre, whereas the third tier (Tier 3) concerns a behind the wheel testing, to determine whether diagnosed cognitive decline can be compensated in real traffic conditions. For the tests conducted within IN LIFE participants underwent all Tiers.

This protocol is stepwise and modular. The overall AGILE assessment procedure involves three tiers. A pre-screening and screening are performed in a quiet room in the first tier (Tier 1). In the second Tier (Tier 2) persons failing the screening battery undergo an in-depth diagnostic neuropsychological assessment at a specialised centre, whereas the third tier (Tier 3) concerns a behind the wheel testing, to determine whether diagnosed cognitive decline can be compensated in real traffic conditions. For this study participants underwent all Tiers.

#### First tier: Pre- screening assessment

The first tier of the adapted procedure was a pre- and screening stage where the assessment can be performed to predict the risk of driving difficulties experienced by older drivers. Administration of this screening battery is easy and short, taking less than 20 min, and it contributes to determine which drivers need to continue the driving ability assessment procedure and thus should be referred to an in-depth assessment centre.

The screening battery is composed of three tools. Visuo-spatial attention is measured by the Trail Making Test part A (TMT-A)^18^, executive processing is targeted through a questionnaire of Instrumental Activities of Daily Living (IADL)^20^ and the Executive Control of the TAP-M neuropsychological battery standardized for driving performance^21^. The TAP-M assesses in a dynamic way integrated aspect of cognitive functions relevant to driving, so that compensation can occur. A global final screening score can be computed.

#### Second tier: In-depth neuropsychological assessment

People who fail the screening battery may have impairments resulting in unsafe driving behaviour and driving performance. In order to examine whether this is really the case, they will be referred to the second stage of the procedure, i.e., the in-depth assessment, for a more thorough and detailed cognitive evaluation. In contrast to the pre-screening and screening phase, which is predictive of future driving performance, the in-depth assessment is a diagnostic investigation. The purpose of the neuropsychological test battery is to evaluate in an analytical way the different cognitive functions relevant to safe driving. The Test for Attention Performance—Mobility (TAP-M) by Zimmermann and Fimm^21^, specifically standardized for driving behaviour was administered. The subtests measure the following main functions: non-spatial attention (alertness, active central visual field, Go/ Nogo sub-testes), visuo-spatial attention (active peripheral visual field, visual scanning sub-tests) and executive attention (distractability, divided attention, flexibility). When a participant obtains scores that are above the recommended thresholds (cut-off scores) for each neuropsychological subtest parameter of the extracted factors, then the person was automatically be considered as fit to drive.

#### Third tier: In-depth ‘behind-the-wheel’ assessment

The assessments carried out at the first two tiers focus on possible deficits in cognitive functions relevant for driving. Given that computerized tests and paper-and-pencil tools generally have only a limited predictive value for a person’s actual driving performance, a practical driving test is recommended in case of diagnosed deficits.

Practical driving tests have a higher validity because compensation strategies and personal driving habits can counterbalance moderate reductions in functional skills. More ecological evaluations allow seeing the extent to which compensation occurs, and hence allow determining recommendations in terms of restrictions, adaptations and/or conditions, if needed, and this based on objective criteria.

The outcome of the in-depth NP assessment is the starting point for the third-tier practical testing. Tailoring the on-road evaluation to the results of preceding assessment stages has several advantages. The high face validity of the on-road driving evaluation will make it easier to explain the outcome of the on-road assessment to the evaluated driver. The procedure is at the same time highly efficient: the selection of directly relevant driving tasks and traffic situations minimizes duration and costs for each on-road test.

The on-road test is a pre-defined standardized course, comprised of four different modules, that can be independently assessed. The maximum duration of the on-road test is about 40 min, which would mean that the participant’s performance was unsuccessful at the three NP modules, and thus the four on-road modules had to be performed. In case of a positive result at a given NP module, then the corresponding on-road module can be skipped.

The first module is an independent basic driving skills evaluation module, which has to be performed always before the other modules. The aim of this module is to allow the assessor to judge whether a driver reaches the minimal performance level needed to continue the driving test without endangering road safety. The three other modules are related to the NP factors. Each module targets more specifically one factor of the Neuro Psychological (NP) assessment battery and includes specific traffic situations—known to be particularly critical for the older drivers—whose mastery principally relies on the focused factor. Specific test scenarios include crossing of various intersection conditions (left, right turns, and straight forward drives—at controlled, uncontrolled intersections), including visually complex sections.

The three different on-road evaluation modules associated with the AGILE NP test battery and the general one are the following: *cluster A:* Basic driving skills. *cluster B:* Non-spatial attention, *cluster C:* Visuo-spatial attention, and *cluster D:* Executive attention.

#### Passenger car vehicle and testing route

The passenger can vehicle was a driving instructor vehicle with additional pedals at the co-driver’s seat. A researcher was always present at the backseat of the vehicle. All participants completed the same route. The route was chosen because it fulfils the requirement of the driving test categories and because of its proximity to CERTH premises where the rest of the experiments took place. All participants completed the same route that lasted approximately 40 min. The route is not heavily affected by traffic congestion and, thus, time of driving (e.g., if it is during peak times) does not affect the duration and effort/resources required by drivers.

It was decided to use the passenger car vehicle for the following reasons:

- Real experience cannot -at least for now- be truly emulated.
- Older drivers have increased risk to suffer from driving simulator sickness.
- The driving simulator has automatic, not braking box and although the driving experience is easier it does not reflect the driving population (only 2% of our participants drive an automatic box vehicle).
- The protocol was set up in order to be feasible in both real and simulated driving experience and has already been validated for administration to older drivers.

The whole session lasted approximately 3 h including informed consent and debriefing. Participants completed the driving test the day after completing Tier 1, Tier 2 and olfactory tests to ensure they were not getting tired. All participants received compensation for their participation.

### Olfactory tests

The sniffin’ sticks were used for three separate olfactory tests: (a) threshold test, (b) discriminating test, (c) identification test. The Sniffin’ Sticks test (Burghardt®, Wedel, Germany) is a psychophysical test developed by Hummel in 1997 and validated in several European countries. It allows semi-objective assessment of the patient's olfactory performance by means of 3 subtests: threshold test, identification test and discrimination test. Although it is difficult to perform the test completely and systematically in routine clinical practice, it is one of the essential tools to assess an individual's olfactory performances and to monitor the course of these performances as a function of physiological (ageing) or pathological events.

The Sniffin Sticks are used to investigate human olfactory performance. They have been developed in close cooperation with the association ‘Olfaktologie und Gustologie’ of the German Society for Ear, Nose and Throat Medicine, Head and Neck Surgery (http://www.hno.org/olfaktologie). The Extended Test (also called ‘TDI-Test’—Threshold, Discrimination, Identification) consists of sub-tests with which the ability to identify and discriminate smells is tested and a subtest to test the smell threshold. The complete test takes 30–40 min. The results of all participants are added up to get the so called “TDI-score”. The result allows a detailed assessment of the olfactory function and can also be used in medico-legal cases. Since it is a subjective test procedure, an objective olfactometry is advisable if aggravation or simulation is suspected. The overall olfactory score with the Sniffin’ Sticks battery have found to be able to predict MCI with good sensitivity and specificity (70.3% and 77.4% respectively)^31^.

#### Threshold test

The smell threshold is determined in a so called “staircase procedure”. After a start concentration of the smell is found out, the dilution step is identified at which the smell can just be distinguished from non-smelling pens (blanks). The Threshold Test was set with n-butanol as target.

#### Discrimination test

Discrimination of odours is based on comparison of 3 smell presentations (triplet). To this end, two times the same odour is presented (non-target) and one time a different smelling one (target). The participant’s task is to indicate which one smells different. This comparison is performed for 16 triplets.

#### Identification test

Here the ability to identify everyday smells by means of a card with 4 choices is determined. It is a multiple-choice procedure, which means the patient has to pick one of the 4 terms. Altogether, 16 odours are presented in the Identification Test.

### Statistical analysis

Statistical analyses were conducted with SPSS (version 22; IBM Corp., Armonk, NY). The significance level was set at *p* < 0.05. Repeated MANOVAs were performed to investigate the mean differences among the three groups across phases and they were performed to identify the variables that can be predictive of the driver’s fitness to drive. and post-hoc (with Bonferroni adjusted *p*-values) tests were used to compare differences between the three groups (MCI and comorbidities, and controls). Only the variables with significant differences among the groups were added in stepwise logistic regressions separately for each Tier, i.e.: ΤΜΑ-Α, Ability index of IADL, tiredness index of IADL, executive errors (from computerized TAP-M test), olfactory threshold, olfactory discrimination, olfactory identification for Tier 1 and for Tier 2. We used binary logistic regression was used to evaluate whether the olfactory and other significant variables predicted their fitness to drive. The logistic regression models were built of the 3 markers (olfactory discrimination, olfactory identification, alertness) controlling for age, sex and education. Tests were bidirectional because we were not sure if any deviations would be found in older active drivers, although we would expect that lower scores would be found for MCI participants based on literature.

## Data Availability

The data that support the findings of this study are available from the Horizon 2020 EU project IN LIFE but restrictions apply to the availability of these data, which were used under license for the current study, and so are not publicly available. Data are however available from the authors upon reasonable request and with permission of the IN LIFE project. The person responsible for providing the data upon request is Katerina Touliou.
